# Inhibitory Pedunculopontine Neurons Gate Dopamine-Mediated Motor Actions of Unsigned Valence

**DOI:** 10.2174/1570159X21666230911103520

**Published:** 2023-09-11

**Authors:** Sirin Zhang, Juan Mena-Segovia, Nadine K. Gut

**Affiliations:** 1 Center for Molecular and Behavioral Neuroscience, Rutgers University, Newark, NJ, USA

**Keywords:** Pedunculopontine, GABA, dopamine, action selection, reward, threat, avoidance, axon terminals

## Abstract

**Background:**

The pedunculopontine nucleus (PPN) maintains a bidirectional connectivity with the basal ganglia that supports their shared roles in the selection and execution of motor actions. Previous studies identified a role for PPN neurons in goal-directed behavior, but the cellular substrates underlying this function have not been elucidated. We recently revealed the existence of a monosynaptic GABAergic input from the PPN that inhibits dopamine neurons of the substantia nigra. Activation of this pathway interferes with the execution of learned motor sequences when the actions are rewarded, even though the inhibition of dopamine neurons did not shift the value of the action, hence suggesting executive control over the gating of behavior.

**Objective:**

To test the attributes of the inhibition of dopamine neurons by the PPN in the context of goal-directed behavior regardless of whether the outcome is positively or negatively reinforced.

**Methods:**

We delivered optogenetic stimulation to PPN GABAergic axon terminals in the substantia nigra during a battery of behavioral tasks with positive and negative valence.

**Results:**

Inhibition of dopamine neurons by PPN optogenetic activation during an appetitive task impaired the initiation and overall execution of the behavioral sequence without affecting the consumption of reward. During an active avoidance task, the same activation impaired the ability of mice to avoid a foot shock, but their escape response was unaffected. In addition, responses to potential threats were significantly attenuated.

**Conclusion:**

Our results show that PPN GABAergic neurons modulate learned, goal-directed behavior of unsigned valence without affecting overall motor behavior.

## 1 INTRODUCTION

Goal-directed behavior is the selection and execution of actions that are intended to lead to favorable outcomes: attaining a positive outcome like finding food or avoiding a negative outcome like a potential threat. The basal ganglia have long been recognized as the central hub for action selection, integrating cognitive and motor functions through its complex circuitry of afferent and efferent connectivity [1-4]. An important modulator of basal ganglia function is dopamine [5-9]. Decades of research have implicated dopamine and its effects on striatal activity as a major player in action selection through its role in encoding the initiation and termination of behavioral sequences [10-15] in reward-associated learning by providing a reinforcement signal (*i.e*., the reward prediction error) [16-20], and in encoding choice it self [21, 22]. To understand how dopamine activity is modulated across distinct behavioral contexts, it is essential to identify the major sources of excitation and inhibition to dopamine neurons and characterize their impact on dopamine function.

One such major source is the pedunculopontine nucleus (PPN), which provides dopamine neurons with a rich and dense array of afferents, including cholinergic, glutamatergic and GABAergic [23-27]. Similar to the basal ganglia, the PPN has been shown to encode motor and cognitive functions. Glutamatergic neurons have been identified as a central component of the mesencephalic locomotor region due to their capability to initiate or stop locomotion and adjust muscle tone [28-32]. Cholinergic neurons have been proposed to mediate adaptive behavior by signaling a deviation from expected associations due to changing contingencies [33-37]. Both cholinergic and glutamatergic neurons modulate the activity of dopamine neurons and the release of dopamine across striatal regions [23, 38-41]. In contrast to the excitatory neurons, GABAergic neurons are less well-characterized. Nevertheless, we have recently reported that their most prominent axonal target is the substantia nigra pars compacta, where they inhibit dopamine neurons and block goal-directed behavioral sequences [27].

The role of PPN neurons in goal-directed behavior has previously been investigated. Inactivation or lesioning of the PPN led to failure in goal-directed tasks that required a change of behavioral strategy. When faced with a change of contingencies, increase of lever pressing demands or reward degradation, rats failed to adapt their actions and made perseverative errors [42-44]. Furthermore, tetrode recordings in freely moving mice showed that PPN neurons encode previous choices and therefore influence later decisions, which are impacted by PPN inactivation [45]. PPN neurons have also been shown to respond to cues and rewards [46-50] and encode motivational value and salience [51-53]. Altogether, these studies suggest a prominent role of PPN neurons in adaptive behavior through their influence over the selection of shifting goals and the execution of selected behavioral responses. However, the mechanistic basis for such functions has not been elucidated.

Based on the dense connectivity of PPN GABAergic (PPN_GABA_) neurons over dopamine neurons and their influence on reinforced behavior [27], we set out to fully characterize the impact of PPN inhibition on dopamine-mediated goal-directed behavior and identify what specific aspects of the behavioral sequence are affected. If PPN-mediated inhibition of dopamine neurons reduced the value of a reward, we would expect to find an effect only on positively reinforced behavior. However, if PPN inhibition regulated the expression of behavior by adjusting the motor response to the expected valence of the action, we would expect to observe a behavioral effect regardless of whether the outcome leads to a reward or prevents punishment/threat (negative reinforcement). Our results show that under opposite reinforcement contingencies, PPN_GABA_ neurons gate the expression of purposive behavior and suggest a universal role for the PPN to direct the choice of behavior depending on the context.

## 2 MATERIALS AND METHODS

### 2.1 Animals

All experimental procedures were approved by Rutgers University’s Institutional Animal Care and Use Committee (IACUC) and in accordance with the standards outlined in the eighth edition of the Guide for the Care and Use of Laboratory Animals (National Academy of Sciences, The National Academies Press, Washington, D.C.). Adult male and female (>3 months old) VGAT::Cre mice (Jackson Laboratory, 028862), in which the Cre recombinase expression is associated with the vesicular GABA transporter, were used in all experiments. All animals were single-housed and maintained on a 12:12 light cycle (light on at 7 am), with *ad libitum* access to water. Food restriction was implemented for one of the behavioral tasks (see below).

### 2.2 Surgical Procedures

General anesthesia was induced and maintained with Isofluorane while animals were secured in a stereotaxic frame (Kopf Instruments). Body temperature was maintained at 37 ± 1°C with a heating pad. All surgical tools were sterilized with an autoclave or heat bead sterilizer. A small incision was made on the scalp, exposing the skull surface. Burr holes were drilled in the skull at the designated stereotaxic coordinates for PPN (AP and ML relative to Bregma, DV relative to dura: -4.3 AP, ± 1.2 ML, -3.4 DV). Viral constructs were infused using a microsyringe (Hamilton Company) connected to an electronic pump at a rate of 5nl/min and 40 nl of AAV-EF1a-DIO-hChR2(H134R)-EYFP-WPRE-pA or AAV-EF1a-DIO-EYFP-WPRE-pa (control virus) were injected bilaterally into the PPN for experimental and control animals, respectively. Animals were housed under BSL-2 quarantine conditions and received postoperative care for 3 days post-surgery. After 3 days, they were moved to the BSL-1 area for continued monitoring.

Following the viral injection surgeries, animals were allowed to recover for at least two weeks before undergoing chronic implantation of optic fibers above the substantia nigra pars compacta to target PPN_GABA_ axons. The anesthetic and postoperative procedures were identical to those described above. Burr holes were drilled at the designated stereotaxic coordinates (AP and ML relative to Bregma, DV relative to dura): -3.1 AP, ± 1.5 ML, -3.6 DV. Mice were then implanted bilaterally with custom-made 200 μm-diameter optic fibers (fiber and ferrules Thorlabs), 200μm above the substantia nigra. The implants were secured with skull screws (McMaster Carr) and dental cement (Prime-Dent).

### 2.3 Behavioral Procedures

#### 2.3.1 Open Field Evaluation with Anymaze

**Fig. (1):**
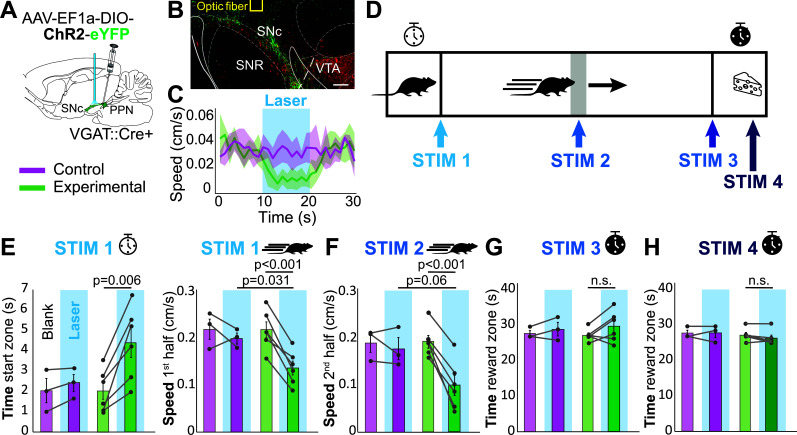
Stimulation of PPN_GABA_ axons in the substantia nigra affects action initiation and execution but not consumptive behavior. (**A**) Schematic description of the experimental preparation. (**B**) Histological verification of the optic fiber placement above the substantia nigra and adjacent to YFP-positive PPN axons expressing ChR2. (**C**) Optogenetic stimulation in the open field reduced spontaneous locomotor activity (as in [27]) and was used as the inclusion criterion for subsequent experiments. (**D**) Runway design: all animals started the trials in the starting zone. The total length of the runway was divided into two equally long halves and contained a reward zone at the opposite end of the runway. Stimulation was delivered at one out of four possible locations along the runway (STIM 1, 2, 3 and 4; order counterbalanced). (**E**) In STIM 1 trial, experimental animals spent significantly longer in the starting zone and reduced their speed in the first half of the runway. (**F**) STIM 2 significantly reduced the speed of mice in the second half of the runway. (**G**) STIM 3 caused an increase in time spent in the reward zone (from entering to finishing reward consumption) in a subset of animals but did not reach significance. (**H**) STIM 4 did not affect reward consumption as measured by the time spent in the reward zone. **Abbreviations:** SNc, substantia nigra pars compacta; SNr, substantia nigra pars reticulata. Scale bar in B: 150 µm. Datapoints in E-H represent individual animals. Data represented as mean ± SEM.

The animals were allowed at least six weeks after the injection surgeries and two weeks after the implantation surgeries before they participated in behavioral tasks. All mice were handled by the experimenter in the animal facility for 10 min, twice per day for 3 days before testing. They were then subjected to a single-trial, open-field session to test the efficacy of the optic stimulation [27]. Animals were brought to the experimental room and allowed 20 min of room acclimation in their home cages. During the trial, a split patch cord (Thorlabs) was connected to the implanted optic fiber ferrules. Optic stimulation (10 s pulse train: 20 Hz, 20 ms) was provided by a blue laser (473 nm, CrystaLaser) connected to the patch cord *via* a rotary joint (Doric Lenses Inc). To ensure a constant stimulation power across trials and animals, the laser output was titrated with an optical power monitor (Thorlabs) before each session to be at 6 mW with the above stimulation parameters. Once an animal was attached to the patch cord, it was released into an open field arena (40 x 40 cm) and its behavior was tracked with ANY-maze tracking software (Stoelting Co.). Each trial consisted of the following: 5 min of free exploration without laser stimulation, followed by 10 min of stimulation epochs (30s epochs as follows: 10s laser off, 10s laser on and 10s laser off; (n = 20 epochs per trial; (Fig. **1**, **1C**)). After the testing phase, animals were returned to their home cages.

#### 2.3.2 Runway Task

Prior to this task, mice were food restricted to increase motivation during training. Training on the runway task commenced once the animals reached 85% of their baseline bodyweight. The runway is an elongated rectangular arena (64 cm* 11.5 cm* 12.5 cm), with a gate at one end blocking access to the rest of the runway. All trials started in the starting zone (8 cm* 11.5 cm* 12.5 cm) with the gate closed. At the other end of the runway, the reward zone (8 cm* 11.5 cm *12.5 cm) consistently contained one piece of food reward during all trials. To acclimate animals to the runway, mice were placed in the starting zone for 2 min, after which the gate opened, and they were allowed free exploration for 10 min. A food reward (white chocolate chip) was placed at the end of the runway (in the reward zone) as reinforcement. During the training phase, every animal underwent four non-consecutive trials per session. After 1 min in the starting zone, the gate opened, and the mouse was allowed to navigate to the reward zone in order to consume the food reward. If any of the following behaviors took place, the mouse was immediately taken out of the runway and placed in the home cage as a time-out punishment: (1) if it did not exit the starting zone in 10 s, (2) if it paused for more than 1s in the runway, (3) if it started to walk back to the starting zone while in the runway, or (4) if it did not start eating within 3 s of arriving at the reward zone. If animals received a time-out punishment, they did not have an opportunity to make up for the missed trial. Each day, one randomly selected trial was run with the patch cords attached to the optic fiber implants for habituation. Animals moved to the testing phase once they completed four consecutive unpunished trials. The testing phase lasted for 14 days, and each day the animals underwent four trials. For two of the trials, laser stimulation was not delivered (blank trials), and the trials served as within-group controls. For the two experimental trials (laser trials), laser stimulation was administered for 5 seconds at 4 possible time points: (1) immediately after the starting zone gate opened and the animal gained access to the runway (STIM 1), (2) when the animal crossed the midpoint of the runway (STIM 2), (3) when the animal reached the end of the runway before entering the reward zone (STIM 3), or (4) five seconds after the animal entered the reward zone (STIM 4; (Fig. **1**, **1D**). The four types of laser trials were counterbalanced, and the animal velocity and position were recorded using the ANYmaze software. All trials were manually monitored and terminated immediately after the animal finished the food reward so that the time spent in the food zone could be used as a representation of time spent consuming the food reward.

#### 2.3.3 Active Avoidance Task

**Fig. (2):**
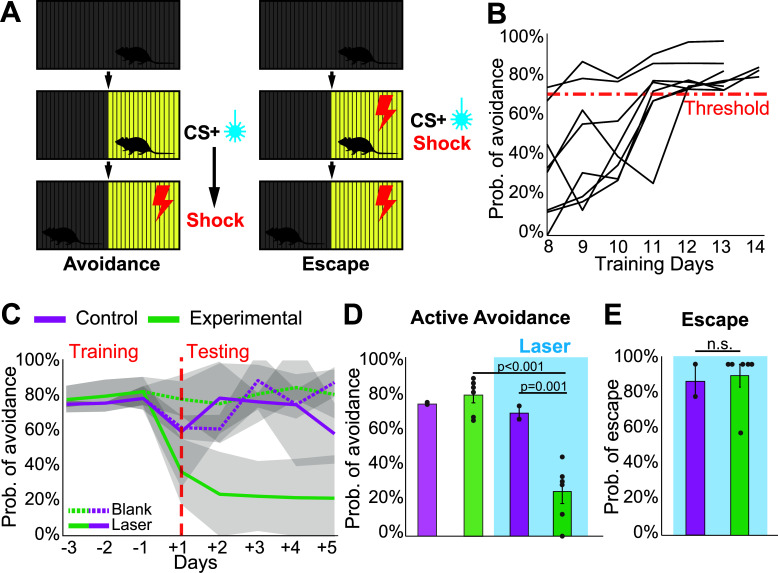
Stimulation of PPN_GABA_ axons in the substantia nigra impaired active avoidance but not escape behavior. (**A**) Active avoidance trials consisted of the presentation of a conditioned stimulus (CS, light) followed by a shock on the same side of the chamber after a 5s delay. In 50% of the trials, the CS was paired with optogenetic stimulation. Escape trials consisted of a mild foot shock paired with laser stimulation (*i.e*., without preceding CS). (**B**) Mice learned the active avoidance task within 13-14 days (each line represents one animal). (**C**, **D**) Experimental animals performed significantly worse than controls during the laser trials in the active avoidance task. No difference between experimental and control animals was observed during blank trials. The performance of control animals remained consistent between blank and laser trials. The shaded area in D represents SEM. (**E**) No difference in the ability to escape the foot shock was observed between groups during laser trials. Datapoints in D and E represent individual animals. Data represented as mean ± SEM.

Animals entered the training phase of the active avoidance task only after they fully recovered their baseline body weight from food restriction. For two consecutive days, the animals were habituated to the experimental room and testing chamber. The active avoidance chamber consisted of an open arena (60 cm* 28.5 cm) divided into two identical halves, each containing an individual grid tile. Each grid tile was connected to a separate shocker capable of delivering weak electrical currents through the connected tile only. LED light panels were placed under each grid tile to serve as visual stimuli (Fig. **2A**). After acclimation, the training phase began. Each day the animals underwent one training session consisting of 3 min of acclimation followed by 54 trials of the active avoidance task. Each trial started with one of the two light panels lighting up for 5 s, which served as the conditioned stimulus (CS). After 5 s of CS presentation, a weak electric shock (0.4 mA) was delivered through the metal grid above the lit panel as the unconditioned stimulus (US). The shock lasted for 5s, during which the light panel stayed on. After the 10s CS presentation, both the light panel and the shocker were turned off, and animals had an intertrial interval (ITI) of 25-35 s before the next trial commenced. To preclude side biases, each session was pseudorandomized so that the left and right grid tiles delivered the CS + US combination for an equal number of times. During training, an active avoidance trial was considered successful when the animal started the trial on the lit grid tile but managed to avoid the electric shock by moving to the other grid tile. If the animal started the trial on the unlit tile and moved into the lit tile during the shock, the trial was discarded. Training performance was determined by the number of active avoidance responses made. Once the animals reached the threshold of 70% successful active avoidance performance for 3 consecutive days, they were moved to the testing phase. One animal was not able to reach the threshold and was excluded from the experiment.

The active avoidance testing protocol was identical to the training protocol, with the addition of a 5 s laser stimulation (20 Hz, 20 ms pulses; 473 nm) time-locked to the onset of the CS in 50% of the trials. Animal performance under stimulation was recorded with the ANYmaze system for analysis. The testing phase lasted for five days, after which one single session of escape testing (*i.e*., unavoidable shock) was performed. During this session, the light and the shock were presented concomitantly for five seconds and were followed by a 25-35 s ITI. The laser stimulation was present in every trial and was time-locked to the onset and offset of the shock. The trials were pseudorandomized so that the left and right tiles were activated an equal number of times, and the session concluded after the animal received 10 shocks.

#### 2.3.4 Novel Object Interaction Task

Mice were first habituated to being connected to the patchcord for optogenetic stimulation in an open field over at least two 15 min sessions. Subsequently, they were habituated to a box of 30*45 cm with a white floor, white walls, and no objects for 15 min. General locomotor activity was measured during the habituation sessions. After habituation, a novel object (a torch) was placed in the center of the box, and we measured the animals’ interactions with the object in a 15-min session. When the animals entered within a 50mm radius of the novel object (head first, entries of other parts of the body did not count), a blue laser for optogenetic stimulation was activated. The pulse train was the same as described above. When mice left this stimulation zone, the laser stopped. If they stayed for longer than 10 s, the laser paused for a duration of 5 s and resumed again unless the mice had left the zone by then. We measured the time mice spent within this radius and only considered the time their heads were oriented towards the object as “interaction with the object”, to rule out the possibility that mice intended to retreat but were too slow or unable to do so.

### 2.4 Immunohistochemistry and Histological Verification of Injection and Implantation Site

Following the completion of all experiments, animals were euthanized with an intraperitoneal injection of pentobarbital solution (250 mg/kg) and perfused with 0.1 M phosphate buffer solution (PBS) followed by 4% paraformaldehyde solution. Brains were removed, and sagittal sections were collected using a vibrating microtome (Leica) at 50 μm thickness. Sections selected for staining were 300 μm apart on the mediolateral axis. Immunohistochemical processing was initiated by blocking the sections with 10% normal donkey serum (Jackson Immunoresearch) in PBS-Triton for 1.5 h. Primary antibodies against choline acetyltransferase (host: goat, 1:500, AB144P, Millipore), tyrosine hydroxylase (host: mouse, 1:1000, T2928, Sigma; or host: rabbit, 1:500, AB152, Millipore), and green fluorescent protein (conjugated with Alexa 488; host: rabbit, 1:1000, A21311, Invitrogen) were incubated overnight. Secondary antibodies conjugated with different fluorophores were incubated on the following day for 3.5 h (Cy5, anti-goat 705-175-147, anti-rabbit 711-175-152, anti-mouse 715-175-150; Alexa405, anti-rabbit 711-475-152; all 1:250, Jackson Immunoresearch). Sections were then rinsed with PBS and mounted with a mounting medium (Vectashield). Sections containing the PPN were used for the anatomical verification of the expression of ChR2 in PPN_GABA_ neurons. Cholinergic neurons identified by the presence of a positive reaction to Anti-ChAT served as the approximate boundary of the PPN. Sections containing the SNc were assessed for the location of the optic fibers. The fiber tip had to be 200-400 um above the SNc, identified by TH-positive neurons.

### 2.5 Statistical Analysis

The significance level was set at *P* ≤ 0.05. Parametric testing was used whenever possible to test differences between two or more means. Normality was tested using the Shapiro-Wilk tests, and Levene’s test was conducted to assess homogeneity. Mild violations of normality and homogeneity were accepted. For severe violations of normality and homogeneity in the active avoidance task, the non-parametric Mann-Whitney-U test was used. Main effects and interactions were followed up by planned comparisons when found significant and Bonferroni corrected. Statistical tests were done using SPSS (IBM) and Matlab (MathWorks).

## 3 RESULTS

### 3.1 Inhibition of Dopamine Neurons by PPN_GABA_ Axons Blocks Specific Components of a Reinforced Behavioral Sequence

We have previously shown that inhibiting dopamine neurons by activating PPN_GABA_ afferents interrupts goal-directed action sequences in an operant task [27]. To determine whether the inhibitory effect interferes with the overall engagement in goal-directed behavior or with specific components of it (*i.e*., the initiation, execution of the task or consumption of the reward), we tested mice in an appetitive conditioning task that allows the parcellated analysis of the motor sequence. For this purpose, we expressed ChR2 in PPN_GABA_ neurons of VGAT::Cre mice and implanted optic fibers above the substantia nigra (Fig. **1A**, **B**). Before training, mice were tested in the open field to confirm the previously reported decrease in exploratory locomotion during PPN_GABA_ axon stimulation [27]; only mice that showed the described response to the laser were included in the experimental group (mixed ANOVA, interaction: F(1,7) = 54.55, *p <* 0.001, n = 9; univariate ANOVA, control *vs.* experimental: F(1,7) = 41.678, *p <* 0.001, control: n = 3, experimental: n = 6; Fig. **1C**).

Next, we trained the mice in a custom-made runway task where animals learned to traverse a corridor without interruption to retrieve a food reward at the opposite end (see Methods for details). To analyze the performance of mice during this task, the runway was segmented into 4 zones: the starting zone (closed by a gate) to evaluate action initiation, the first and second halves of the runway to evaluate action execution and vigor, and the reward zone to evaluate reward retrieval and consummatory behavior. Mice learned to complete the task following an average of 34.44 ± 0.88 trials, taking an average of 3.78s ± 1.44s in each trial. Following training, we tested mice by delivering a 5s-long, bilateral optogenetic stimulation train through the implanted optic fibers at the following locations along the runway: in the starting zone at gate opening (STIM 1) to test the stimulation effect on action initiation, at the midpoint of the runway (STIM 2) to test ongoing action execution, at the end of the runway before entering the reward zone (STIM 3) to test the stimulation effect on action transition from running to reward retrieval, and in the reward zone to test the effect on reward consumption (STIM 4; Fig. **1D**). We found that optogenetic stimulation in experimental animals (n = 6) during STIM 1 trials delayed the initiation of the motor action, and they remained significantly longer in the starting zone than the controls (n = 3; Fig. **1E** left; mixed ANOVA, interaction: F(1,7) = 6.676, p = 0.036, n = 9 [sphericity assumed]; repeated measures ANOVA on experimental group: F(1,5) = 21.573, *p* = 0.006, n = 6). Interestingly, STIM 1 trials also produced an effect on the vigor of the execution of the task, shown by a significantly slower speed in experimental animals in the first half of the runway (Fig. **1E** right; mixed ANOVA, interaction: F(1,7) = 7.029, *p* = 0.033, n = 9 (sphericity assumed); repeated measures ANOVA on the experimental group: F(1,5) = 49.208, *p <* 0.001, n = 6). Similarly, we found that during STIM 2 trials (stimulation at the midpoint of the runway), action execution was affected, as shown by a significant reduction in the speed of experimental animals (Fig. **1F**); mixed ANOVA, interaction: F(2,14) = 5.118, *p* = 0.021, n = 9 (sphericity assumed); repeated measure ANOVA on experimental group: F(2,10) = 19.518, *p <* 0.001, n = 6). STIM 3 trials tested if the stimulation affects the completion of the task (reaching the end of the runway) when mice transition from running to retrieving the reward. The combined time mice spent in the reward zone from entering to the end of food consumption was notably increased in 3 out of 6 experimental mice (Fig. **1G**). However, no differences were observed between groups in STIM 4 trials, suggesting that PPN-mediated inhibition of dopamine activity does not interfere with reward retrieval and consumption (Fig. **1H**); mixed ANOVA, interaction: F(2,14) = 0.779, *p* = 0.478, n = 9 (sphericity assumed); repeated measure ANOVA on the experimental group: F(2,10) = 4.825, *p* = 0.034, n = 6 (sphericity assumed); *post hoc* Bonferroni corrected: blank *vs*. Stim 3: *p* = 0.402; blank *vs*. Stim 4: *p* = 0.278; Stim 3 *vs* Stim 4: *p* = 0.143). These results demonstrate that the inhibitory effect of PPN_GABA_ neurons on dopamine neurons delays action initiation and reduces the vigor of the execution of goal-directed behavior but does not have an effect on innate, consumptive behavior.

### 3.2 Optogenetic Activation of PPN_GABA_ Axons Impairs Active Avoidance Behavior

Dopamine neurons have been shown to encode a variety of aversive stimuli and contribute critically to the generation of goal-directed behavioral sequences to avoid threat or harm [54-56]. We, therefore, aimed to determine whether learned actions in response to stimuli signaling aversive outcomes are also delayed or interrupted by dopamine inhibition by PPN_GABA_ axons. Mice were trained in an active avoidance paradigm in which they were conditioned to avoid a mild foot shock by crossing to the alternative side of the testing chamber following the presentation of a visual cue (see Methods for details; Fig. **2A**). During the training phase, active avoidance performance for all animals steadily improved until it reached a pre-defined 70% threshold (Fig. **2B**). During the testing phase, the onset of the conditioned stimulus (light) was paired with PPN_GABA_ axon stimulation in the substantia nigra in 50% of the trials. We found that the stimulation prevented experimental animals (n = 6) from engaging in avoidance behavior, *i.e*., they did not cross over to the alternative side of the chamber as control animals did (n = 2) and therefore received a mild foot shock (Fig. **2C**). Accordingly, the percentage of successful active avoidance trials significantly dropped in experimental animals when stimulated (Fig. **2D**; 2x2 (trial type x group) 2-way ANOVA, interaction: F(1,12) = 11.573, *p* = 0.005; control *vs* experimental group: F(1,12) = 18.574, *p* = 0.001, n = 8). This effect did not cause any extinction of the learned behavior nor a decrease in their willingness and motivation to complete the task: during the blank trials (no laser delivery), the experimental animals performed at a level (80% ± 14%) that is comparable both to their own pre-testing performance (80% ± 8%) and that of the control animals (75% ± 15%; mixed ANOVA, interaction: F(1,6) = 0.04, *p* = 0.85, n = 8). In a small percentage of trials (‘escape’ trials), mice received a foot shock simultaneously with laser stimulation without the conditioned stimulus (*i.e*., the light and stimulation were presented concomitantly with the foot shock). In contrast to the active avoidance trials, the behavior during escape trials was not affected: experimental animals escaped the shock-paired side of the chamber in less than 5 seconds (*i.e*., before the end of the optogenetic stimulation) in almost all the trials, and their success rate in escaping did not differ from controls (Mann-Whitney U test: U = 7.5, z = 0.655, *p* = 0.643, n = 8; Fig. **2E**). These results suggest that under negative reinforcement contingencies, motor actions are gated by PPN_GABA_ neurons. Together with the previous results in the appetitive runway task, our data show that PPN_GABA_ neurons engage in modulating action responses of both appetitive and aversive valence, suggesting a role in gating goal-directed behavior under salient contingencies.

### 3.3 Activation of PPN_GABA_ Neurons Reduces Retreat Behavior Without Prior Experience of Negative Outcomes

**Fig. (3):**
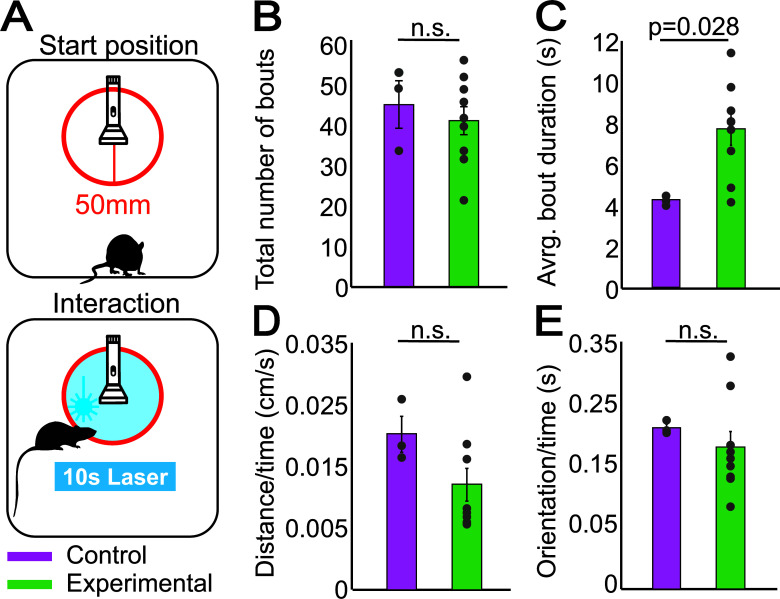
Stimulation of PPN_GABA_ axons in the substantia nigra diminishes retreat responses in a novel object interaction task. (**A**) Optogenetic stimulation was delivered when mice entered a radius of 50mm within the novel object. Stimulation duration was either 10s or until mice left the 50mm radius. (**B**, **C**) Experimental animals engaged in the same number of interaction bouts as control animals but had significantly longer interaction bouts with the novel object. (**D**, **E**) Optogenetic stimulation neither reduced distance traveled nor changed head orientation towards the object in experimental animals, suggesting that the increased interaction time in C was not due to motor impairment. Datapoints in B and E represent individual animals. Data represented as mean ± SEM.

Previous studies have shown that exploratory behavior of novel objects is organized in bouts of approaches followed by avoidance behavior (*i.e*., retreats) and that avoidance responses to novel stimuli and potential threats are blocked by ablating dopamine neurons projecting to the so-called tail of the striatum (TS) [54, 57]. Further, dopamine neurons that encode stimulus intensity and value project to the caudal end of the striatum, including the TS [58]. Because PPN_GABA_ neurons innervate the lateral substantia nigra and their activation decreases dopamine release in the caudal part of the striatum [27], we set out to investigate whether PPN_GABA_ neurons are capable of modulating avoidance responses to novel objects. To this end, mice were exposed to a novel object in a testing cage, and their location with respect to the object was recorded during the trial. When mice entered within a 50 mm radius of the object (stimulation zone), and their head was oriented towards it, optogenetic stimulation was delivered for 10 s (or until the animals exited the 50 mm radius; stimulation parameters as described above; Fig. **3A**). The stimulation did not cause the experimental mice (n = 9) to engage in more or fewer bouts of approach-avoidance compared to control mice (n = 3; univariate ANOVA, control *vs* experimental group: F(1,10) = 0.302, *p* = 0.595, n = 12; Fig. **3B**). However, in comparison to control animals, experimental animals interacted for a longer time with the novel object, therefore suggesting less engagement in retreat behavior (Fig. **3C**; univariate ANOVA, control *vs*. experimental group: F(1,10) = 6.603, *p* = 0.028, n = 12). Importantly, the stimulation did not produce a motor effect that reduced their ability to retreat, as both groups moved similarly within the stimulation zone (Fig. **3D**; univariate ANOVA, control *vs*. experimental group: F(1,10) = 2.706, *p* = 0.131, n = 12) and experimental mice exited the stimulation zone on average after 7.7s ± 0.8s SEM (*i.e*., before the laser was off; laser duration: 10s). Furthermore, experimental mice were oriented towards the novel object for as long as controls did (Fig. **3E**; univariate ANOVA, control *vs*. experimental group: F(1,10) = 0.474, *p* = 0.507, n = 12), suggesting a similar type of *active* engagement with the object while animals were inside the zone. Overall, experimental animals increased the interaction time with the object from the first visit, as opposed to controls which gradually increased their interactions by presumably learning that the object posed no threat (data not shown). These data suggest that dopamine neurons receiving inhibitory input from the PPN have a role in the initiation, potentially through reinforcement [54], of avoidance behavior, which the PPN is able to modulate. Our data thus show that the inhibitory input from the PPN to dopamine neurons is capable of modulating learned, goal-directed behaviors, not only when associated with a reward or punishment but even if the positive outcome (avoidance of a threat) is innate.

## 4 DISCUSSION

The results presented here demonstrate that PPN_GABA_ neurons participate in the modulation of goal-directed behavior through their connectivity with dopamine neurons of the substantia nigra. We first showed that PPN_GABA_-mediated inhibition of dopamine neurons perturbed discrete elements of a goal-directed behavioral sequence, delaying the initiation and execution (vigor) of actions without affecting reward consumption. Next, we showed that the same experimental manipulation impaired the ability of animals to initiate conditioned avoidance behavior following the presentation of a cue that predicts a foot shock, whereas the escape motor response was unaffected. Last, we showed that activation of PPN_GABA_ neurons decreases the perception of threat associated with a novel object, leading to more engagement with, and less retreat from, the object. Taken together, our results suggest that PPN_GABA_ neurons interfere with the integration of sensory cues that predict positive or negative outcomes, therefore effectively blocking the learned actions necessary to reach a goal (approach or avoid). Interestingly, however, we recently showed that activation of PPN_GABA_ neurons also blocks the initiation and execution of self-paced reinforced behavior that is not associated with any cues [27], suggesting that rather than a failure to associate a cue with an outcome, PPN_GABA_ neurons specifically block actions that are modulated by their valence, regardless of whether these are positively or negatively reinforced (leading to a reward or the removal of a threat; *i.e*., unsigned). These effects are in agreement with the role of dopamine over striatal regions associated with the initiation of actions [59-62], the modulation of action vigor [63-66] and the encoding of valence [67-69].

Striatal dopamine signals are critical for the initiation and execution of goal-directed action sequences. Both striatal projection neurons (SPNs) and dopamine neurons of the substantia nigra increase their firing rate prior to the initiation of goal-directed behavior the disruption of which leads to delay or abortion of planned behavior [10]. Optogenetic inhibition of dopamine neurons decreases the probability of action initiation of learned sequences [12], while both optogenetic excitation and inhibition of SPNs increase latency to action initiation [70, 71]. These behavioral observations resemble the effects that we showed on the runway, where stimulation of the PPN_GABA_ terminals before action initiation (starting chamber) significantly delayed learned behavior, likely through the inhibition of dopamine release [27] and the subsequent perturbation of SPN activity. During action execution, such as lever pressing, dopamine levels increase [72], and direct pathway SPNs maintain an elevated firing rate extending over the entire motor sequence [11]. A decrease in striatal dopamine levels changes the balance between the activity of direct and indirect SPNs and biases the basal ganglia output towards the indirect pathway, interrupting ongoing actions, possibly in favor of alternative actions [71]. By inhibiting dopamine release in the striatum [27], activation of PPN_GABA_ neurons may be shifting the striatal output in favor of the activation of indirect SPNs.

By stimulating PPN_GABA_ axons in the lateral part of the substantia nigra (where PPN axons show their densest distribution), dopamine neurons projecting to the caudal parts of the dorsolateral striatum (including the TS) were most likely inhibited [73]. Dopamine activity in these striatal areas encodes stimuli of positive value but also stimulus intensity, including stimuli that signal potential threats and lead to avoidance behavior [54, 74, 75]. The effect of inhibition of dopamine neurons by the PPN on the initiation and execution of the runway task supports the effect of a reduction of dopamine release in the dorsolateral striatum [10, 11, 76]. In contrast, the behavioral effects observed in the active avoidance and novel object interaction tasks suggest a reduction of dopamine release in the TS [54, 57]. Dopamine in the TS suppresses engagement with a novel object, and the ablation of TS-projecting dopamine neurons inhibits avoidance behavior, like a retreat from a novel stimulus [54]. The prolonged interaction time with the novel object and the reduction of foot shock avoidance resemble the effects of TS ablations in the literature. Our data thus suggest that PPN inputs recruit specific circuits within the dopaminergic midbrain that will modulate striatal functions across distinct regions. Future experiments will aim to elucidate whether midbrain inhibition by PPN neurons generates a wide-ranging reduction in dopamine that simultaneously affects multiple striatal regions or whether specialized midbrain circuits are recruited based on their input-output connectivity.

## CONCLUSION

Evidence from earlier work, together with the above findings, show the ability of the PPN to disrupt ongoing behaviors or stop goal-directed actions from being initiated directly *via* its inhibitory projections to the dopamine midbrain. Behavioral sequences during spontaneous exploratory behavior can also be stopped by the PPN in favor of alternative behaviors. Goal-directed behaviors of distinct associative contingencies can be interrupted: reward-associated actions and threat-related behaviors (with and without previous association with a negative outcome) that are essential for an adapted interaction with the environment and, ultimately, survival. This suggests a modulatory role of the PPN in the decision-making process where several alternative actions are possible and governed by the potential outcome of the action on two axes: the value of the outcome (positive or negative) and the potential associated risk attached to the action that would lead to that outcome. This could mean interrupting reward-related actions if some alternative action can lead to an outcome of higher value or biasing risk assessment towards engagement with a new stimulus instead of avoidance because this might increase the chances of finding food, for example. This pathway might be relevant for symptoms such as bradykinesia and freezing of gait in Parkinson’s disease, maladapted risk-seeking behaviors, and the development of phobias.

## Data Availability

Not applicable.

## LIST OF ABBREVIATIONS

CS: Conditioned Stimulus
PBS: Phosphate Buffer Solution
PPN: Pedunculopontine Nucleus
SPNs: Striatal Projection Neurons
US: Unconditioned Stimulus

## References

[1] Marsden C.D. (1982). The mysterious motor function of the basal ganglia: The Robert Wartenberg Lecture.. Neurology.

[2] Yin H.H. (2017). The basal ganglia in action.. Neuroscientist.

[3] Hikosaka O., Kim H.F., Yasuda M., Yamamoto S. (2014). Basal ganglia circuits for reward value-guided behavior.. Annu. Rev. Neurosci..

[4] Dudman J.T., Krakauer J.W. (2016). The basal ganglia: From motor commands to the control of vigor.. Curr. Opin. Neurobiol..

[5] Redgrave P., Rodriguez M., Smith Y., Rodriguez-Oroz M.C., Lehericy S., Bergman H., Agid Y., DeLong M.R., Obeso J.A. (2010). Goal-directed and habitual control in the basal ganglia: Implications for Parkinson’s disease.. Nat. Rev. Neurosci..

[6] Hikosaka O., Ghazizadeh A., Griggs W., Amita H. (2018). Parallel basal ganglia circuits for decision making.. J. Neural Transm. (Vienna).

[7] Schultz W. (2016). Reward functions of the basal ganglia.. J. Neural Transm. (Vienna).

[8] Rice M.E., Patel J.C., Cragg S.J. (2011). Dopamine release in the basal ganglia.. Neuroscience.

[9] Haber S.N. (2014). The place of dopamine in the cortico-basal ganglia circuit.. Neuroscience.

[10] Jin X., Costa R.M. (2010). Start/stop signals emerge in nigrostriatal circuits during sequence learning.. Nature.

[11] Jin X., Tecuapetla F., Costa R.M. (2014). Basal ganglia subcircuits distinctively encode the parsing and concatenation of action sequences.. Nat. Neurosci..

[12] da Silva J.A., Tecuapetla F., Paixão V., Costa R.M. (2018). Dopamine neuron activity before action initiation gates and invigorates future movements.. Nature.

[13] Bakhurin K.I., Li X., Friedman A.D., Lusk N.A., Watson G.D.R., Kim N., Yin H.H. (2020). Opponent regulation of action performance and timing by striatonigral and striatopallidal pathways.. eLife.

[14] Kravitz A.V., Freeze B.S., Parker P.R.L., Kay K., Thwin M.T., Deisseroth K., Kreitzer A.C. (2010). Regulation of parkinsonian motor behaviours by optogenetic control of basal ganglia circuitry.. Nature.

[15] Bartholomew R.A., Li H., Gaidis E.J., Stackmann M., Shoemaker C.T., Rossi M.A., Yin H.H. (2016). Striatonigral control of movement velocity in mice.. Eur. J. Neurosci..

[16] Schultz W., Dayan P., Montague P.R. (1997). A neural substrate of prediction and reward.. Science.

[17] Watabe-Uchida M., Eshel N., Uchida N. (2017). Neural circuitry of reward prediction error.. Annu. Rev. Neurosci..

[18] Lerner T.N., Holloway A.L., Seiler J.L. (2021). Dopamine, updated: Reward prediction error and beyond.. Curr. Opin. Neurobiol..

[19] Cohen J.Y., Haesler S., Vong L., Lowell B.B., Uchida N. (2012). Neuron-type-specific signals for reward and punishment in the ventral tegmental area.. Nature.

[20] Tsutsui-Kimura I., Matsumoto H., Uchida N., Watabe-Uchida M. (2020). Distinct temporal difference error signals in dopamine axons in three regions of the striatum in a decision-making task.. ELife.

[21] Parker N.F., Cameron C.M., Taliaferro J.P., Lee J., Choi J.Y., Davidson T.J., Daw N.D., Witten I.B. (2016). Reward and choice encoding in terminals of midbrain dopamine neurons depends on striatal target.. Nat. Neurosci..

[22] Moss M.M., Zatka-Haas P., Harris K.D., Carandini M., Lak A. (2021). Dopamine axons in dorsal striatum encode contralateral visual stimuli and choices.. J. Neurosci..

[23] Galtieri D.J., Estep C.M., Wokosin D.L., Traynelis S., Surmeier D.J. (2017). Pedunculopontine glutamatergic neurons control spike patterning in substantia nigra dopaminergic neurons.. eLife.

[24] Lavoie B., Parent A. (1994). Pedunculopontine nucleus in the squirrel monkey: Cholinergic and glutamatergic projections to the substantia nigra.. J. Comp. Neurol..

[25] Clarke P.B.S., Hommer D.W., Pert A., Skirboll L.R. (1987). Innervation of substantia nigra neurons by cholinergic afferents from pedunculopontine nucleus in the rat: Neuroanatomical and electrophysiological evidence.. Neuroscience.

[26] Gould E., Woolf N.J., Butcher L.L. (1989). Cholinergic projections to the substantia nigra from the pedunculopontine and laterodorsal tegmental nuclei.. Neuroscience.

[27] Gut N.K., Yilmaz D., Kondabolu K., Huerta-Ocampo I., Mena-Segovia J. (2022). Selective inhibition of goal-directed actions in the mesencephalic locomotor region.. BioRxiv.

[28] Josset N., Roussel M., Lemieux M., Lafrance-Zoubga D., Rastqar A., Bretzner F. (2018). Distinct contributions of mesencephalic locomotor region nuclei to locomotor control in the freely behaving mouse.. Curr. Biol..

[29] Roseberry T.K., Lee A.M., Lalive A.L., Wilbrecht L., Bonci A., Kreitzer A.C. (2016). Cell-type-specific control of brainstem locomotor circuits by basal ganglia.. Cell.

[30] Caggiano V., Leiras R., Goñi-Erro H., Masini D., Bellardita C., Bouvier J., Caldeira V., Fisone G., Kiehn O. (2018). Midbrain circuits that set locomotor speed and gait selection.. Nature.

[31] Dautan D., Kovács A., Bayasgalan T., Diaz-Acevedo M.A., Pal B., Mena-Segovia J. (2021). Modulation of motor behavior by the mesencephalic locomotor region.. Cell Rep..

[32] Masini D., Kiehn O. (2022). Targeted activation of midbrain neurons restores locomotor function in mouse models of parkinsonism.. Nat. Commun..

[33] Gut N.K., Mena-Segovia J. (2022). Midbrain cholinergic neurons signal negative feedback to promote behavioral flexibility.. Trends Neurosci..

[34] Dautan D., Huerta-Ocampo I., Gut N.K., Valencia M., Kondabolu K., Kim Y., Gerdjikov T.V., Mena-Segovia J. (2020). Cholinergic midbrain afferents modulate striatal circuits and shape encoding of action strategies.. Nat. Commun..

[35] MacLaren D.A.A., Markovic T., Clark S.D. (2014). Assessment of sensorimotor gating following selective lesions of cholinergic pedunculopontine neurons.. Eur. J. Neurosci..

[36] Ruan Y., Li K.Y., Zheng R., Yan Y.Q., Wang Z.X., Chen Y., Liu Y., Tian J., Zhu L.Y., Lou H.F., Yu Y.Q., Pu J.L., Zhang B.R. (2022). Cholinergic neurons in the pedunculopontine nucleus guide reversal learning by signaling the changing reward contingency.. Cell Rep..

[37] Xiao C., Cho J.R., Zhou C., Treweek J.B., Chan K., McKinney S.L., Yang B., Gradinaru V. (2016). Cholinergic mesopontine signals govern locomotion and reward through dissociable midbrain pathways.. Neuron.

[38] Blaha C.D., Winn P. (1993). Modulation of dopamine efflux in the striatum following cholinergic stimulation of the substantia nigra in intact and pedunculopontine tegmental nucleus-lesioned rats.. J. Neurosci..

[39] Dautan D., Souza A.S., Huerta-Ocampo I., Valencia M., Assous M., Witten I.B., Deisseroth K., Tepper J.M., Bolam J.P., Gerdjikov T.V., Mena-Segovia J. (2016). Segregated cholinergic transmission modulates dopamine neurons integrated in distinct functional circuits.. Nat. Neurosci..

[40] Yoo J.H., Zell V., Wu J., Punta C., Ramajayam N., Shen X., Faget L., Lilascharoen V., Lim B.K., Hnasko T.S. (2017). Activation of pedunculopontine glutamate neurons is reinforcing.. J. Neurosci..

[41] Estakhr J., Abazari D., Frisby K., McIntosh J.M., Nashmi R. (2017). Differential control of dopaminergic excitability and locomotion by cholinergic inputs in mouse substantia nigra.. Curr. Biol..

[42] Wilson D.I.G., MacLaren D.A.A., Winn P. (2009). Bar pressing for food: Differential consequences of lesions to the anterior versus posterior pedunculopontine.. Eur. J. Neurosci..

[43] MacLaren D.A.A., Wilson D.I.G., Winn P. (2013). Updating of action–outcome associations is prevented by inactivation of the posterior pedunculopontine tegmental nucleus.. Neurobiol. Learn. Mem..

[44] Taylor C.L., Kozak R., Latimer M.P., Winn P. (2004). Effects of changing reward on performance of the delayed spatial win-shift radial maze task in pedunculopontine tegmental nucleus lesioned rats.. Behav. Brain Res..

[45] Thompson J.A., Costabile J.D., Felsen G. (2016). Mesencephalic representations of recent experience influence decision making.. eLife.

[46] Okada K., Kobayashi Y. (2015). Rhythmic firing of pedunculopontine tegmental nucleus neurons in monkeys during eye movement task.. PLoS One.

[47] Tian J., Huang R., Cohen J.Y., Osakada F., Kobak D., Machens C.K., Callaway E.M., Uchida N., Watabe-Uchida M. (2016). Distributed and mixed information in monosynaptic inputs to dopamine neurons.. Neuron.

[48] Skvortsova V., Palminteri S., Buot A., Karachi C., Welter M.L., Grabli D., Pessiglione M. (2021). A causal role for the pedunculopontine nucleus in human instrumental learning.. Curr. Biol..

[49] Norton A.B.W., Jo Y.S., Clark E.W., Taylor C.A., Mizumori S.J.Y. (2011). Independent neural coding of reward and movement by pedunculopontine tegmental nucleus neurons in freely navigating rats.. Eur. J. Neurosci..

[50] Okada K., Toyama K., Inoue Y., Isa T., Kobayashi Y. (2009). Different pedunculopontine tegmental neurons signal predicted and actual task rewards.. J. Neurosci..

[51] Hong S., Hikosaka O. (2014). Pedunculopontine tegmental nucleus neurons provide reward, sensorimotor, and alerting signals to midbrain dopamine neurons.. Neuroscience.

[52] Pan W.X., Hyland B.I. (2005). Pedunculopontine tegmental nucleus controls conditioned responses of midbrain dopamine neurons in behaving rats.. J. Neurosci..

[53] Kobayashi Y., Inoue Y., Yamamoto M., Isa T., Aizawa H. (2002). Contribution of pedunculopontine tegmental nucleus neurons to performance of visually guided saccade tasks in monkeys.. J. Neurophysiol..

[54] Menegas W., Akiti K., Amo R., Uchida N., Watabe-Uchida M. (2018). Dopamine neurons projecting to the posterior striatum reinforce avoidance of threatening stimuli.. Nat. Neurosci..

[55] Ungless M.A., Argilli E., Bonci A. (2010). Effects of stress and aversion on dopamine neurons: Implications for addiction.. Neurosci. Biobehav. Rev..

[56] Matsumoto M., Hikosaka O. (2009). Two types of dopamine neuron distinctly convey positive and negative motivational signals.. Nature.

[57] Tsutsui-Kimura I., Uchida N., Watabe-Uchida M. (2022). Dynamical management of potential threats regulated by dopamine and direct- and indirect-pathway neurons in the tail of the striatum.. bioRxiv.

[58] Poulin J.F., Caronia G., Hofer C., Cui Q., Helm B., Ramakrishnan C., Chan C.S., Dombeck D.A., Deisseroth K., Awatramani R. (2018). Mapping projections of molecularly defined dopamine neuron subtypes using intersectional genetic approaches.. Nat. Neurosci..

[59] Ko D., Wanat M.J. (2016). Phasic dopamine transmission reflects initiation vigor and exerted effort in an action- and region-specific manner.. J. Neurosci..

[60] Augustin S.M., Loewinger G.C., O’Neal T.J., Kravitz A.V., Lovinger D.M. (2020). Dopamine D2 receptor signaling on iMSNs is required for initiation and vigor of learned actions.. Neuropsychopharmacology.

[61] Wassum K.M., Ostlund S.B., Maidment N.T. (2012). Phasic mesolimbic dopamine signaling precedes and predicts performance of a self-initiated action sequence task.. Biol. Psychiatry.

[62] Markowitz J.E., Gillis W.F., Jay M., Wood J., Harris R.W., Cieszkowski R., Scott R., Brann D., Koveal D., Kula T., Weinreb C., Osman M.A.M., Pinto S.R., Uchida N., Linderman S.W., Sabatini B.L., Datta S.R. (2023). Spontaneous behaviour is structured by reinforcement without explicit reward.. Nature.

[63] Beierholm U., Guitart-Masip M., Economides M., Chowdhury R., Düzel E., Dolan R., Dayan P. (2013). Dopamine modulates reward-related vigor.. Neuropsychopharmacology.

[64] Mazzoni P., Hristova A., Krakauer J.W. (2007). Why don’t we move faster? Parkinson’s disease, movement vigor, and implicit motivation.. J. Neurosci..

[65] Mohebi A., Pettibone J.R., Hamid A.A., Wong J.M.T., Vinson L.T., Patriarchi T., Tian L., Kennedy R.T., Berke J.D. (2019). Dissociable dopamine dynamics for learning and motivation.. Nature.

[66] Zénon A., Devesse S., Olivier E. (2016). Dopamine manipulation affects response vigor independently of opportunity cost.. J. Neurosci..

[67] Van Wouwe N.C., Claassen D.O., Neimat J.S., Kanoff K.E., Wylie S.A. (2017). Dopamine selectively modulates the outcome of learning unnatural action-valence associations.. J. Cogn. Neurosci..

[68] Koob G.F. (1996). Hedonic valence, dopamine and motivation.. Mol. Psychiatry.

[69] Hamid A.A., Pettibone J.R., Mabrouk O.S., Hetrick V.L., Schmidt R., Vander Weele C.M., Kennedy R.T., Aragona B.J., Berke J.D. (2016). Mesolimbic dopamine signals the value of work.. Nat. Neurosci..

[70] Cui G., Jun S.B., Jin X., Pham M.D., Vogel S.S., Lovinger D.M., Costa R.M. (2013). Concurrent activation of striatal direct and indirect pathways during action initiation.. Nature.

[71] Tecuapetla F., Jin X., Lima S.Q., Costa R.M. (2016). Complementary contributions of striatal projection pathways to action initiation and execution.. Cell.

[72] Syed E.C.J., Grima L.L., Magill P.J., Bogacz R., Brown P., Walton M.E. (2016). Action initiation shapes mesolimbic dopamine encoding of future rewards.. Nat. Neurosci..

[73] Menegas W., Bergan J.F., Ogawa S.K., Isogai Y., Umadevi Venkataraju K., Osten P., Uchida N., Watabe-Uchida M. (2015). Dopamine neurons projecting to the posterior striatum form an anatomically distinct subclass.. eLife.

[74] Akiti K., Tsutsui-Kimura I., Xie Y., Mathis A., Markowitz J.E., Anyoha R., Datta S.R., Mathis M.W., Uchida N., Watabe-Uchida M. (2022). Striatal dopamine explains novelty-induced behavioral dynamics and individual variability in threat prediction.. Neuron.

[75] Gangarossa G., Castell L., Castro L., Tarot P., Veyrunes F., Vincent P., Bertaso F., Valjent E. (2019). Contrasting patterns of ERK activation in the tail of the striatum in response to aversive and rewarding signals.. J. Neurochem..

[76] Crego A.C.G. (2020). Štoček, F.; Marchuk, A.G.; Carmichael, J.E.; van der Meer, M.A.A.; Smith, K.S. Complementary control over habits and behavioral vigor by phasic activity in the dorsolateral striatum.. J. Neurosci..

